# A comparison of intimate partner and other sexual assault survivors’ use of different types of specialized hospital-based violence services

**DOI:** 10.1186/s12905-017-0408-9

**Published:** 2017-08-07

**Authors:** Janice Du Mont, Maryam Woldeyohannes, Sheila Macdonald, Daisy Kosa, Linda Turner

**Affiliations:** 1 Women’s College Research Institute, Women’s College Hospital, 76 Grenville Street, 6th Floor, Toronto, Ontario M5S 1B2 Canada; 2 Dalla Lana School of Public Health, University of Toronto, Toronto, Ontario Canada; 3 Ontario Network of Sexual Assault/Domestic Violence Treatments Centres, Toronto, Ontario Canada

**Keywords:** Sexual assault, Assailant, Intimate partner, Acquaintance, Stranger, Service use

## Abstract

**Background:**

Little is known about the health service utilization of women sexually assaulted by their intimate partners, as compared with those sexually assaulted by other perpetrators. To address this gap, we describe the use of acute care services post-victimization, as well as a broad range of survivor and assault characteristics, across women assaulted by current or former intimate partners, other known assailants, and strangers.

**Methods:**

Information was gathered from individuals presenting to 30 hospital-based sexual assault and domestic violence treatment centres using a standardized data collection form. We examined the data from 619 women 16 years of age or older who were sexually assaulted by one assailant.

**Results:**

Women sexually assaulted by a current or former intimate partner were less likely than those assaulted by another known assailant or a stranger to have been administered emergency contraception (*p* < 0.001) or prophylaxis for sexually transmitted infections (*p* < 0.001), and counselled for potential use of HIV post-exposure prophylaxis (*p* < 0.001). However, these women were more likely than those in the other two groups to have had their injuries documented with photographs (*p* < 0.001), have undergone a risk assessment (*p* = 0.008), and/or have engaged in safety planning (*p* < 0.001).

**Conclusions:**

Women sexually assaulted by current or former intimate partners utilized services offered by sexual assault and domestic violence treatment centres differently than those assaulted by other known assailants and strangers. This may reflect their different health, forensic, and social needs, as well as the importance of offering care tailored to their particular circumstances.

**Electronic supplementary material:**

The online version of this article (doi:10.1186/s12905-017-0408-9) contains supplementary material, which is available to authorized users.

## Background

The high prevalence of sexual assault, documented worldwide, largely can be attributed to pervasive cultures of male dominance that cultivate stringent gender inequalities and contribute to a pernicious rape discourse fuelled by rape myths [1, 2]. Disturbingly, the harmful victimization of sexual assault survivors is often discredited or trivialized by rape myths which include attitudes and generally false beliefs about who is a “legitimate” victim and skewed perceptions of what constitutes a “legitimate” rape [3, 4]. These myths are also often drawn upon to justify the perpetration of sexual assault and defend the assailant’s actions [3, 4]. Examples of rape myths include the following: women lie about rape, women who drink or wear sexy clothes deserve to be raped, and rape is a crime of passion [3, 4]. Identifying and unpacking such myths is crucial as, according to Suarez and Gadalla and other researchers of violence, “the concept of rape myths contribute in a significant way to the understanding of rape and its consequences to victims” [5], p. 2013.

One of the most pernicious myths about sexual assault is that it is an act committed by a stranger. However, more than two decades of research has shown that most sexual assaults are committed by known assailants—a substantial proportion of which are committed by intimate partners, a grouping which in research typically has included current or former spouses (married or common-law), boyfriends, girlfriends, and/or other dating partners (e.g., [6–12]). In the most recent and largest study to date conducted on intimate partner and sexual violence in the United States, more than 50% of women who reported having been raped sometime during their lifetime indicated that at least one assailant was a current or former intimate partner [6].

There is a persistent and popular misconception that sexual assaults perpetrated by intimate partners are less serious than those perpetrated by strangers. However, some recent research has shown the opposite to be true [11, 12]. In a recent study by Möller and colleagues, for example, women sexually assaulted by an current or former intimate partner were significantly more likely than those assaulted by a stranger to have experienced severe assaults with vaginal (80% vs. 54%), anal (25% vs. 6%), and oral (25% vs. 12%) rape and extreme forms of violence, including having been strangled (21% vs. 8%), kicked (19% vs. 3%), and subjected to multiple forms of physical assault (49% vs. 29%) [11].

Another popular myth is that sexual assaults perpetrated by intimate partners have less severe health consequences than those perpetrated by strangers. Currently available research shows this not always to be true; sexual assaults committed by intimate partners are often similar to or graver than those committed by strangers in terms of the consequences [12, 13]. For example, Murphy and colleagues found that those women sexually assaulted by a current or former intimate partner were more likely to experience genital injuries (29% vs. 15%) than those sexually assaulted by a stranger [12]. Survivors of sexual assault by an intimate partner also have been shown to experience psychological symptoms at rates higher than survivors of sexual assaults by non-intimate partner assailants, including post-traumatic stress disorder, dissociation, and stress [14] and be almost twice as likely to suffer from depression or anxiety [15].

Societal and individual adherence to these myths or misconceptions may impact the help-seeking of survivors of sexual assault by an intimate partner [16]. There is some evidence to indicate that survivors of sexual assault by intimate partners may be less likely than those assaulted by strangers to report to the police [17] and seek help from crisis intervention services [18]. In one small, older study, Koss and colleagues found that 19% of stranger rape, 3% of non-romantic acquaintance rape, 3% of casual date rape, 1% of steady date rape, and 0% of ‘spouse-family’ rape survivors sought help from crisis counselling and support services at the time of a sexual assault [18].

In North America, and increasingly in other countries around the world, acute health care services following a sexual assault are delivered by Sexual Assault Nurse Examiners (SANE), who are specially trained in the collection and documentation of forensic evidence and provision of sexual assault medical care [19]. Evaluations of these programs have found that survivors of sexual assault use a wide range of the services offered [20], and report that these services are delivered in a caring and sensitive manner that addresses their acute needs [21]. However, there has been little investigation of the use of these services by women sexually assaulted by different types of assailants, which could be critical to better understanding the needs of those assaulted by their intimate partners who are known to have an increased risk of revictimization [11].

This gap in the literature is noteworthy as in some of the few studies examining survivors of intimate partner sexual assault who access these services the uptake of different aspects of care appears to be different than among survivors of sexual assault by other assailants. Although comparisons across studies must be made cautiously as the categories used and definitions for known assailants have varied, in one such study of 331 women who were examined after presenting in the emergency department at an urban community hospital in the United States, Logan and colleagues found that women sexually assaulted by a current or former intimate partner were more likely than those assaulted by an acquaintance, acquaintance just met, or stranger to have had photographs taken for evidence [13]. Similarly, in a study by Stermac, Du Mont, and Dunn of 1162 women presenting to a sexual assault centre in Toronto, Ontario those assaulted by a current or former intimate partner were more likely to have had a sexual assault evidence kit (also known as rape kit) completed for potential court use than those assaulted by an acquaintance known for less than 24 hours, an acquaintance known for more than 24 hours, and a stranger [22]. To build on this sparse research and better understand the use of SANE-led acute care services by women sexually assaulted by an intimate partner, we examined information collected from survivors presenting to Ontario’s hospital-based Sexual Assault/Domestic Violence Treatment Centre (SA/DVTC)s as part of a larger project focused on a client evaluation of services (see [21]). Our objectives were to describe the use of acute care services as well as a broad range of survivor and assault characteristics across women assaulted by current or former intimate partners, other known assailants, and strangers. This information could aid in the identification of any potential gaps in service utilization for these women and inform discussion of whether the services offered may be sensitive to their specific needs.

## Methods

Thirty hospital-based SA/DVTCs participated in the service evaluation, the methods for which have been described elsewhere [21, 23]. Ethics board approval was obtained at all participating sites. The central coordinating site was Women’s College Hospital (WCH111012007–2008).

### Data collection process

All data were collected prospectively. From April 1, 2009 through June 30, 2011, clients were interviewed by attending SANEs as part of the delivery of clinical care. Each client was offered services as appropriate, including health care (e.g., crisis counselling, medical care/treatment, sexually transmitted infection [STI] prophylaxis, HIV post exposure prophylaxis [PEP] counselling, pregnancy prophylaxis), forensic evaluation (e.g., vaginal examination with speculum, anal/rectal examination, assessment and documentation of injuries, photo documentation of injuries, sexual assault evidence kit completion), risk assessment and safety planning, and referral for ongoing support (e.g., on-site follow-up care and counselling, referral to community agencies for counselling and other services). Clients were free to accept or decline any services. The information about service use was collected from clients who consented to participate in the evaluation using a 28-item standardized intake form completed by the attending SANE who also gathered information about date and time of presentation, and client (e.g., age, marital status, employment status, disability status, living situation, social supports), assailant (e.g., relationship to assailant), and assault (e.g., type of sex act, use of coercive tactics, weapon use, physical injuries) characteristics (see Tables for full listing of variables). The data collection form was designed to collect information potentially desirable from a research standpoint (e.g., variables that have been associated with sexual assault and service utilization in previous studies), while not placing too high a burden on the client during a crisis admission. The intake forms were sent to the central coordinating centre where a research assistant reviewed the forms for clarity and completeness, and entered them into a secure, password-protected database.

### Analysis

In the current study, the analyses included women 16 years of age and older who were sexually assaulted by one assailant for whom the identity was provided. For the bivariate analyses, we collapsed assailant type into three categories allowing for comparisons among our categories of interest: current or former intimate partner (e.g., [ex]husband, [ex]boyfriend), other known assailant (e.g., parent or guardian, other relative, acquaintance, friend, co-worker), and stranger. In the analyses, cross-tabulation with Chi-square analyses were performed to describe the relationship between assailant type and service utilization, as well as time from assault to presentation at hospital, and client and assault characteristics. Where appropriate, Fisher’s exact test was used. Additionally, a multivariate logistic regression analysis examining the potential associations between relationship type and any services use, adjusting for potential confounders, was considered. However, almost 100% of clients in our study had used at least one service, precluding such an approach. A *p* value < 0.05 indicated statistical significance. SPSS Statistics version 20.0 was used for the analyses.

## Results

There were 619 women aged 16 years of age and older who indicated their relationship to the assailant and were assaulted by a single assailant. Current or former intimate partners were responsible for 18.9% of sexual assaults, while the majority of survivors (63.2%) had been sexually assaulted by other known assailants, including friends (15.5%) and acquaintances (39.9%). Strangers were the assailants in 17.9% of cases (see Table 1).

**Table 1:** Assailant-survivor relationship type among women presenting to sexual assault treatment centres in Ontario

Type of relationship	*N* = 619 (%)
Current intimate partner	62 (10.0)
Former intimate partner	55 (8.9)
Parent or guardian	11 (1.8)
Other relative	11 (1.8)
Acquaintance	247 (39.9)
Friend	96 (15.5)
Co-worker	11 (1.8)
Authority figure	7 (1.1)
Sex-trade customer	8 (1.3)
Stranger	111 (17.9)

### Time from assault to presentation at hospital by relationship to assailant

**Fig. 1:**
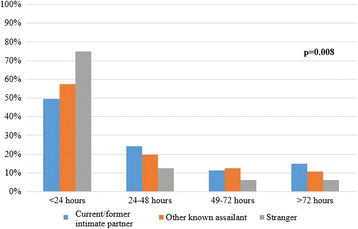
Time from assault to presentation at hospital by assailant-victim relationship type among women presenting to sexual assault treatment centres in Ontario (*n* = 612)

Women sexually assaulted by a current or former intimate partner were more likely to delay seeking treatment as compared with those assaulted by another known assailant or a stranger. They were less likely to present to the hospital within 24 h (49.6% vs 57.3% & 74.8%, respectively; *p* = 0.008) of being sexually assaulted (Fig. 1).

### Client characteristics by relationship to assailant

Women sexually assaulted by a current or former intimate partner differed from other sexual assault survivors on some sociodemographic characteristics. Women sexually assaulted by an intimate partner were older overall than those assaulted by another known assailant or a stranger. They were more likely to be between the ages of 25 and 44 (47.0% vs 32.5% & 33.3%, respectively) and be 45 years of age and older (10.3% vs 4.6% & 4.5%; *p* = 0.004). Women sexually assaulted by an intimate partner were also more likely to be married, living common-law, or cohabiting (29.6% vs 10.1% & 18.0%) and separated, divorced, or widowed (17.4% vs 9.5% and 9.9%), and were less likely to identify as single (53.0% vs 80.4% & 72.1%; *p* < 0.001; see Table 2).

**Table 2:** Client characteristics by assailant-survivor relationship type among women presenting to sexual assault treatment centres in Ontario

Client characteristic	Current/former intimate partner	Other known assailant	Stranger	*p* value
Age group, years	*n* = 117 (%)	*n* = 391 (%)	*n* = 111 (%)	0.004
16–18	21 (17.9)	110 (28.1)	25 (22.5)	
19–24	29 (24.8)	136 (34.8)	44 (39.6)	
25–44	55 (47.0)	127 (32.5)	37 (33.3)	
45+	12 (10.3)	18 (4.6)	5 (4.5)	
Marital status	*n* = 115 (%)	*n* = 388 (%)	*n* = 111 (%)	<0.001
Single	61 (53.0)	312 (80.4)	80 (72.1)	
Separated/divorced/widowed	20 (17.4)	37 (9.5)	11 (9.9)	
Married/common-law/cohabiting	34 (29.6)	39 (10.1)	20 (18.0)	
Employed	*n* = 115 (%)	*n* = 373 (%)	*n* = 102 (%)	0.982
	51 (44.3)	168 (45.0)	45 (44.1)	
Disability	*n* = 117 (%)	*n* = 391(%)	*n* = 111 (%)	0.941
	22 (18.8)	69 (17.6)	19 (17.1)	
Living situation^a^	*n* = 117 (%)	*n* = 380 (%)	*n* = 109 (%)	
Alone	25 (21.4)	73 (19.2)	23 (21.1)	0.832
With family	86 (73.5)	244 (64.2)	67 (61.5)	0.112
With roommate/in dormitory	13 (11.1)	70 (18.4)	24 (22.0)	0.081
Homeless/shelter/institution	10 (8.5)	27 (7.1)	6 (5.5)	0.673
Social supports^a^	*n* = 116 (%)	*n* = 386 (%)	*n* = 109 (%)	
None	12 (10.3)	32 (8.3)	4 (3.7)	0.141
Family	85 (73.3)	320 (82.9)	90 (82.6)	0.061
Friend/roommate/colleague	71 (61.2)	257 (66.6)	75 (68.8)	0.443
Mental health/community/school	34 (29.3)	115 (29.8)	32 (29.4)	0.993

^a^Categories are not mutually exclusive

### Assault characteristics by relationship to assailant

Women sexually assaulted by a current or former intimate partner experienced different assaults than women assaulted by another known assailant or a stranger. They were more likely to have been vaginally (65.8% vs 56.3% & 49.5%, respectively; *p* = 0.043) and anally (13.7% vs 6.6% & 9.0%; *p* = 0.054) penetrated with a penis, although the latter finding only approached statistical significance. They were also more likely to have been physically coerced (80.7% vs 49.2% & 58.0%; *p* < 0.001) and verbally threatened or manipulated (43.0% vs 25.7% & 21.0%; *p* < 0.001). As well, they were more likely to have sustained physical injuries as a result of the assault (53.2% vs 32.3% & 33.3%; *p* < 0.001). In contrast, women sexually assaulted by an intimate partner were less likely than those assaulted by another known assailant or a stranger to have been sexually assaulted while asleep or after having been forced to drink alcohol or drugged (11.4% vs 29.4% & 25.0%; *p* = 0.001). Finally, those sexually assaulted by an intimate partner were less likely than those assaulted by another known assailant, but more likely than those assaulted by a stranger, to have had cunnilingus forced upon them (9.4% vs 14.1% & 2.7%; *p* = 0.003; see Table 3).

**Table 3:** Assault characteristics by assailant-survivor relationship type among women presenting to sexual assault treatment centres in Ontario

Assault characteristic	Current/former intimate partner	Other known assailant	Stranger	*p* value
Type of sex acts^a^	*n* = 117 (%)	*n* = 391 (%)	*n* = 111 (%)	
Cunnilingus	11 (9.4)	55 (14.1)	3 (2.7)	0.003
Fellatio	13 (11.1)	59 (15.1)	13 (11.7)	0.434
Completed vaginal penetration (with penis)	77 (65.8)	220 (56.3)	55 (49.5)	0.043
Completed anal/rectal penetration (with penis)	16 (13.7)	26 (6.6)	10 (9.0)	0.054
Tactics used^a^	*n* = 114 (%)	*n* = 354 (%)	*n* = 100 (%)	
None	7 (6.1)	47 (13.3)	13 (13.0)	0.111
Physical violence (restrained/pushed/slapped/beaten/ strangled/stabbed)	92 (80.7)	174 (49.2)	58 (58.0)	<0.001
Verbal coercion (threatened/manipulated)	49 (43.0)	91 (25.7)	21 (21.0)	<0.001
Altered consciousness (sleeping, forced to drink alcohol/drugged)	13 (11.4)	104 (29.4)	25 (25.0)	.001
Weapon	*n* = 110 (%)	*n* = 329 (%)	*n* = 94 (%)	.109
	10 (9.1)	13 (4.0%)	6 (6.4)	
Physical injuries	*n* = 109 (%)	*n* = 378 (%)	*n* = 108 (%)	<0.001
	58 (53.2)	122 (32.3%)	36 (33.3)	

^a^Categories are not mutually exclusive

### Service use by relationship to assailant

Women sexually assaulted by a current or former intimate partner differed from those assaulted by another known assailant or a stranger in their use of certain important acute care services. They were less likely to have received pregnancy prophylaxis (36.8% vs 57.5% & 52.3%, respectively; *p* < 0.001), prophylaxis for STIs (47.9% vs 80.3% & 72.1%; *p* < 0.001), and counselling for potential use of HIV PEP (41.0% vs 70.6% & 65.8%; *p* < 0.001). They were also less likely to have completed a sexual assault evidence kit (55.6% vs 60.9% & 71.2%; *p* = 0.045). In contrast, women sexually assaulted by an intimate partner were more likely to have had their injuries documented with photographs (29.9% vs 13.6% & 11.7%; *p* < 0.001), as well as to have undergone a risk assessment (59.8% vs 43.5% & 48.6%; *p* = 0.008) and safety planning (70.1% vs 41.2% & 41.4%; *p* < 0.001; see Table 4).

**Table 4:** Type of services utilized by assailant-survivor relationship type among women presenting to sexual assault treatment centres in Ontario

Type of service^a^	Current/former intimate partner *n* = 117 (%)	Other known assailant *n* = 391 (%)	Stranger *n* = 111 (%)	*p* value
Assessment and treatment of injuries	103 (88.0)	326 (83.4)	93 (83.8)	0.470
Medical care	87 (74.4)	282 (72.1)	76 (68.5)	0.605
STI prophylaxis	56 (47.9)	314 (80.3)	80 (72.1)	<0.001
HIV PEP counselling	48 (41.0)	276 (70.6)	73 (65.8)	<0.001
Pregnancy prophylaxis	43 (36.8)	225 (57.5)	58 (52.3)	<0.001
Speculum examination	54 (46.2)	206 (52.7)	46 (41.4)	0.077
Anal examination	28 (23.9)	94 (24.0)	27 (24.3)	0.997
Sexual assault evidence kit	65 (55.6)	238 (60.9)	79 (71.2)	0.045
Photo documentation of injuries	35 (29.9)	53 (13.6)	13 (11.7)	<0.001
Crisis counselling	80 (68.4)	250 (63.9)	79 (71.2)	0.308
Risk assessment	70 (59.8)	170 (43.5)	54 (48.6)	0.008
Safety planning	82 (70.1)	161 (41.2)	46 (41.4)	<0.001
On-site follow-up care	92 (78.6)	306 (78.3)	93 (83.8)	0.438
Referral to community services	48 (41.0)	127 (32.5)	42 (37.8)	0.188

Note: *STI* sexually transmitted infection, *PEP* post-exposure prophylaxis

^a^Categories are not mutually exclusive

## Discussion

This study revealed that almost 1 in 5 women presenting to specialized sexual assault and domestic violence treatment services in Ontario had been sexually assaulted by a current or former intimate partner. These findings are consistent with rates of intimate partner sexual assault reported in similar studies, in which survivors had sought medical treatment or care from sexual assault centres, which have ranged from 15% to 18% of the total number of sexual assaults reported [11–13, 22]. As in some earlier research, compared to other sexual assault survivors, victims of intimate partner sexual assault were more likely to experience physical force and injuries, and more serious forms of sexual assault such as having been vaginally and/or anally raped [11–13]. Given these findings, it may be critical for women sexually assaulted by an intimate partner to access acute medical care in a timely manner. However, in this study and others, they have been most likely to delay seeking care [11, 12]. Further outreach and education may be needed to emphasize the importance of accessing services promptly.

In examining the use of a broad range of SA/DVTC services by assailant relationship type, we found that there were several significant differences among women sexually assaulted by a current or former intimate partner and those assaulted by another known assailant or a stranger. Those assaulted by an intimate partner were more likely than those assaulted by another known assailant or a stranger to have had their injuries photo documented for potential release to the police. This may be because these survivors were more likely than other women, as noted in some other studies, to have been subdued using physical force and to have been physically injured [22, 24]. As well, these women were more likely to have used services aimed at decreasing their risk of being revictimized. This care included having undergone an assessment to determine the potential risk for further violent behavior by the perpetrator (e.g., the frequency of violent behavior, any escalation in violent acts, precipitating behaviours such as perpetrator drinking) and having been engaged in appropriate safety planning. These findings are reassuring given that previous research has found that physical and/or sexual assaults committed by intimate partners are often ongoing in nature and can be associated with severe violence, disability, and death, as well as children being in the home who may be exposed to the abuse [25–28].

In contrast, women sexually assaulted by a current or former intimate partner were less likely than those assaulted by another known assailant or a stranger to have received prophylactic treatment at an SA/DVTC for STIs or counselling specifically for their risk of exposure to HIV. This fact may be problematic given that women sexually assaulted by an intimate partner were also potentially at increased risk for some STIs given their higher rates of forced vaginal and anal penetration [28]. However, it is possible that they sometimes chose not to use these services as they did not perceive themselves at risk for STIs because of their familiarity with the assailant. Similarly, it is also possible that providers did not counsel about HIV because they did not consider it appropriate or of value to offer PEP due to the ongoing exposure of the survivor from an intimate assailant [29–32]. In this context, the World Health Organization states that “reducing the ongoing [HIV] risk within the intimate relationship should be emphasized as part of the counselling process” [33], p.8.

Women sexually assaulted by a current or former intimate partner were also less likely than those assaulted by another known assailant or stranger to have been administered emergency medication at a SA/DVTC to prevent an unintended/unwanted pregnancy. This finding is potentially concerning given that women sexually assaulted by an intimate partner were more likely than other sexually assaulted women to have been vaginally penetrated. While it is not clear if the use of emergency contraception was indicated in these women as current contraception use was not documented in this study, previous research has shown that Canadian women in married or in common-law relationships are less likely than other women to be currently using oral contraceptives [34]. Additionally, research specifically focused on women assaulted by intimate partners has shown that they may not be using any form of contraceptive due to fear of repercussions from their intimate partner [35]: “Women who fear or suffer violence from their partners often find it difficult or impossible to discuss contraception with them; they may also fear or suffer abuse if they use contraceptive methods without … permission” [36], p. 8. Therefore, institutional policies should ensure that acute health services discuss and universally offer prophylactic treatment for pregnancy to all survivors of sexual assault, regardless of assailant type [33, 37].

### Limitations

Although this is the first multicentre, province-wide sexual assault study in Canada with a focus on the survivor-assailant relationship and uptake of specialized acute care hospital-based sexual assault services, it has several limitations. As with any large multicentre study, it is possible that data were sometimes collected differently by practitioners across sites. Several steps were taken to limit this possible source of bias including rigorous training of all attending nurses in the use of the standardized data collection forms and regular review by a study coordinator of incoming data for clarity and completeness. Another possible limitation, as with any self-reported outcomes, is the risk of recall and social desirability bias for variables such as those associated with assault characteristics. We worked to limit this bias by training those nurses collecting data to interview clients in an unbiased manner, not to prompt them in their responses to a question, and not to force a “yes” or “no” answer, allowing for an “unsure/unknown” response. Additionally, the study findings may be limited in their generalizability as all data were collected from an acute hospital-based sample which may not be representative of the general population of sexual assault victims given survivors rarely disclose to formal support providers [38]. Finally, future research, conducted in samples with more variability on the use of services post-sexual assault, should include multivariate analyses.

## Conclusions

Although there are widespread sexual assault response programs across Canada and the United States [19], only a handful of studies have examined the use of acute care service utilization by survivor-assailant relationship type. Vigilant attention to and consideration of who are the assailants of sexual assault can help health providers in specialized sexual assault services, as well as other primary care settings, ensure that different survivors’ needs are appropriately addressed by acute care services as well as through prompt referrals to community agencies for other types of support [12, 18]. As survivors of sexual assault by a current or former intimate partner can suffer serious consequences, with more severe violence perpetrated against them than those sexually assaulted by other assailants [11–13, 22, 24], health services responding to these women need to be sure to provide a comprehensive range of care options, particularly those that address the potentially ongoing nature of this type of sexual assault. In any future research examining factors associated with use of services by survivors of sexual assault, the nature of the relationship between the assailant and the survivor is clearly an important factor to consider.

## Additional file

Additional file 1: Ontario Sexual Assault/Domestic Violence Treatment Centres that participated in the Client Evaluation Project. A list of participating centres. (DOCX 14 kb)

## Abbreviations

HIV: Human immunodeficiency virus
PEP: Post exposure prophylaxis
SA/DVTC: Sexual Assault/Domestic Violence Treatment Centre
STI: Sexually transmitted infection
