# Supplementary material for: A comparison of intimate partner and other sexual assault survivors’ use of different types of specialized hospital-based violence services
Source: BMC Womens Health. 2017 Aug 7;17:59. doi: 10.1186/s12905-017-0408-9 (PMC5545831; doi:10.1186/s12905-017-0408-9)
Supplement: Additional file 1: — Ontario Sexual Assault/Domestic Violence Treatment Centres that participated in the Client Evaluation Project. A list of participating centres. (DOCX 14 kb) [file 12905_2017_408_MOESM1_ESM.docx]

**Supplementary File: Ontario Sexual Assault/Domestic Violence Treatment Centres that participated in the Client Evaluation Project**

| **Hospital Name** | **Centre Name** |
| --- | --- |
| Bluewater Health | Sexual & Domestic Assault Treatment Centre |
| Brant Community Healthcare System | Sexual Assault/Domestic Violence Program |
| Brockville General Hospital | Assault Response & Care Centre |
| Chatham-Kent Health Alliance | Sexual Assault/Domestic Violence Treatment Centre |
| Cornwall Community Hospital | Assault and Sexual Abuse Program |
| Dryden Regional Health Centre | Sexual Assault/Domestic Violence Program |
| Grey Bruce Health Services | Sexual Assault & Partner Abuse Care Centre |
| Guelph General Hospital | Guelph-Wellington Care and treatment Centre for Sexual Assault and Domestic Violence |
| Hamilton Health Sciences | Sexual Assault/Domestic Violence Care centre |
| Joseph Brant Hospital | Nina’s Place |
| Kingston General Hospital | Sexual Assault/Domestic Violence Program |
| Lake of the Woods District Hospital | Sexual Assault/Partner Abuse and Safekids Programs |
| Niagara Health System | Sexual Assault /Domestic Violence Treatment Program |
| North Bay General Hospital | Sexual Assault and Domestic Violence Treatment Program |
| Orillia Soldiers’ Memorial Hospital | Regional Sexual & Domestic Assault Program of Simcoe & Muskoka |
| Perth & Smith Falls District Hospital | Lanark County Sexual Assault/Domestic Violence Program |
| Peterborough Regional Health Centre | Sexual Assault/Domestic Violence Program |
| Quinte Health Care | Domestic Violence/Sexual Assault Response Program |
| Renfrew Victoria Hospital | Renfrew Victoria Hospital Regional Assault Care Program |
| Sault Area Hospitals | Sexual Assault/Partner Assault Clinic |
| St. Mary’s General Hospital | Waterloo Region Sexual Assault/Domestic Violence Treatment Centre |
| Sioux Lookout Meno Ya Win Health Centre | Sioux Lookout Assault Care & Treatment Program |
| Sudbury Regional Hospital | Violence Intervention and Prevention Program |
| The Ottawa Hospital | Sexual Assault & Partner Abuse Care Program (SAPACP)/Programme De Soins Aux Victimes D’Agression Sexuelle Et D’abus Par Un Partenaire |
| The Scarborough Hospital | Sexual Assault/Domestic Violence Care Centre |
| Trillium Health Centre | Peel Region Sexual Assault/Domestic Violence Program |
| The Hospital for Sick Children | The Suspected Child Abuse and Neglect (SCAN) Program |
| Thunder Bay Regional Health Sciences Centre | Sexual Assault/Domestic Violence Treatment Centre |
| Women’s College Hospital | Sexual Assault/Domestic Violence Care Centre |
| York Central Hospital | Domestic Abuse & Sexual Assault (DASA) Care Centre of York Region |
